# Development of mucoadhesive adapalene gel for biotherapeutic delivery to vaginal tissue

**DOI:** 10.3389/fphar.2022.1017549

**Published:** 2022-09-29

**Authors:** Hasan Afzaal, Shahiq-uz-Zaman, Adil Saeed, Syed Damin Abbas Hamdani, Amir Raza, Alvina Gul, Mustafeez Mujtaba Babar, Jayakumar Rajadas

**Affiliations:** ^1^ Department of Pharmaceutics, Riphah Institute of Pharmaceutical Sciences, Riphah International University, Islamabad, Pakistan; ^2^ Advanced Drug Delivery and Regenerative Biomaterials, Stanford University School of Medicine, Stanford, CA, United States; ^3^ Department of Basic Medical Sciences, Shifa College of Pharmaceutical Sciences, Shifa Tameer-e-Millat University, Islamabad, Pakistan; ^4^ Department of Chemistry, Gomal University, D. I. Khan, KPK, Pakistan; ^5^ Department of Plant Biotechnology, Atta-ur-Rahman School of Applied Biosciences, National University of Sciences and Technology, Islamabad, Pakistan

**Keywords:** assay development, mucoadhesion, dissolution, intravaginal drug delivery, adapalene

## Abstract

**Purpose:** Alternate formulation strategies need to be devised for improving the absorption and bioavailability of drug molecules administered through the intravaginal route. Enhancing the coating of vaginal mucosa can aid the achievement of this goal. The aim of the current study is to develop a mucoadhesive formulation having adequate adhesiveness, spreading, and viscosity profiles that can ensure good tissue absorption of adapalene upon intravaginal application.

**Method:** A combination of mucoadhesive agents has been employed, including Carbopol-934, HPMC K-15M, and xanthan gum, in varying ratios to formulate five different gels. Furthermore, a cost-effective UV-spectroscopic analytical method was developed to quantify the amount of adapalene in tested samples, both of *in vitro* and *in vivo* origin. The analytical method was validated for different parameters, including specificity, linearity, range, accuracy, precision, and ruggedness. The modified USP-II apparatus was used for dissolution studies, while *in vivo* pharmacokinetic validation was performed in a murine model.

**Result:** Of all the tested formulations, on the basis of the rheo-mechanical attributes, ACX3 performed better than the rest, including the commercially available intravaginal reference product. ACX3 had an average adhesion time of 12 min and a spread diameter of 37 mm. It showed 35 mm as average distance travelled by the diluted sample for leakage assessment. The analytical method developed for the adapalene muco-adhesive gel was within the range for all the validation parameters. For further evaluating the performance of the formulation, dissolution studies were conducted in simulated vaginal conditions which showed 94.83% of drug release within 5 minutes, while on completion of 30 min, it was measured to be 92.90%. Moreover, approximately 67% of the administered drug was recovered after 5 min of administration as evaluated through tissue recovery procedures in mice.

**Conclusion:** The study aided in development of a formulation which can enhance the muco-adhesion of the drug molecule, resulting in an improved pharmacokinetic profile. Moreover, it established an efficient assay method which can be employed for *in vitro* and *in vivo* quantification of adapalene in simulated and physiological fluids.

## 1 Introduction

Adapalene (ADP) belongs to the 3rd generation of retinoids and has been used successfully for treatment of acne. It is known to have comedolytic, anti-inflammatory, immunomodulatory, and anti-proliferative properties (Rusu et al., 2020). In addition to its use in the treatment of dermatological conditions, it has been reported that ADP can potentially target various cancer cell types, including cervical cancer and cervical intraepithelial neoplasia, ovarian cancer, bladder cancer, prostate cancer, and breast cancer (DiSilvestro et al., 2001; Ghosalkar et al., 2018; Rusu et al., 2020; Mehraj et al., 2022b). ADP potentially exhibits its effect by regulating the extracellular signal-regulated protein kinase (ERK1/2) and glutamic-oxaloacetic transaminase 1 (GOT1), thereby causing S-phase arrest, DNA damage, colony formation and migration inhibition, ROS induction, and caspase-mediated apoptosis to ultimately suppress tumor growth (Wang et al., 2019; Mehraj et al., 2022a). However, studies to decipher the exact mechanistic procedures involved in its antineoplastic action are currently underway. As the biological and pharmacological activity of the pharmaceutical ingredient establishes, there is a need to develop pharmaceutically and commercially acceptable formulations. Moreover, the procedures associated with quality control assessment of the drug molecule need to be established so that the formulations can be used for their therapeutic benefits.

The mucoadhesive formulations emerge to be superior in their pharmacotherapeutic properties in comparison to the traditional dosage forms owing to the fact that these bioadhesive formulations ensure longer retention times and better spreadability (Acarturk, 2009). Similarly, intravaginal mucoadhesive dosage forms exhibit improved pharmacokinetic and pharmacodynamic outcomes on account of high perfusion, abundant surface area for absorption, and circumvention of hepatic first-pass effect, thereby ensuring a suitable safety and efficacy profile (Patil et al., 2019). An ideal intravaginal formulation should possess ease of administration in a pain-free manner, thereby ensuring adequate bioavailability and patient compliance (Alawdi and Solanki, 2021). A number of innovative formulations, including vaginal membranes, vaginal films, vaginal rods, and hydrogels, have been developed over the years. Among these, the intravaginal mucoadhesive gels are considered better, primarily due to patient compliance, affluent industrial processing, and pharmacoeconomic value (Vermani et al., 2000). These gels possess the ability to adhere to the mucosal membranes of the female genital tract, thus expansively increasing the residence time and decreasing the administration frequency (Cook and Brown, 2018; Deshkar et al., 2020). The use of mucoadhesive agents, including polysaccharides of natural or synthetic origin, polyacrylates, and cellulose derivatives, has been studied extensively. The optimization of the formulation to achieve the desired characteristics, including appropriate viscosity, to ascertain suitable intravaginal spread and adhesion time is achieved by optimizing the right types and quantities of excipients (Das Neves and Bahia, 2006).

The formulation design of intravaginal mucoadhesive gels of ADP is aimed at assessing the predicted pharmacological response for potential treatment of cervical cancers. In order to ascertain the said response by correlating the pharmacokinetic and pharmacodynamic profiles of ADP, development of an assay method is required that can assist in analyzing the degree of release of the drug from the developed dosage form. Intended at achieving ideal mucoadhesive properties, polymers like Carbopol 934, HPMC K15M, and xanthan gum were used in five (05) different ratios to explore the best combination. The formulations were tested for their mucoadhesive properties such as adhesiveness, spreading potential, leakage, and viscosity. In our current work, a consistent and effective UV-based analytical method has been established for evaluating the release profile of ADP. The best performing formulation was subjected to the developed spectroscopic analysis using the validated method complying the ICH guidelines CPMP/ICH/381/95, 1994, thereby accounting for specificity, linearity, range, accuracy, precision, and ruggedness parameters. The *in vitro* release profile of ADP from the formulated preparation was studied in citrate buffer followed by animal studies for testing the absorption of ADP after intracervical administration in female BALB/c mice. The cervical tissue was analyzed for the absorbed drug over defined time points to ascertain the *in vivo* drug release profile.

## 2 Materials and methods

### 2.1 Standards and reagents

Adapalene was acquired from Everest Organic, India. All raw materials, chemicals, and reagents used, including xanthan gum (Zhongbao Chemicals, China), propylene glycol (Richest Group, Shanghai, China), methyl paraben sodium (Wuhu Huahai Biology Engineering Co. Ltd., China), HPMC-K15M (Hangzhou Zhongbao, China), octasulfonic acid sodium salt (Merck Darmstadt, Germany), phosphoric acid (BDH, France), citric acid (RZBC-JUXIAN Co. Ltd., China), acetonitrile (Merck Darmstadt, Germany), Maylake Blue 1 (Neelikon Food Dyes and Chemicals Ltd., India), HCl 10% solution (RCI Labscan Ltd., Thailand), sodium citrate dihydrate (RZBC-JUXIAN Co. Ltd., China), and reverse osmosis (RO) water (Vitro Diagnostic Laboratories, Pakistan), were of pharmaceutical grade and/or analytical grade.

### 2.2 Instrumentation

The adhesion test was performed on a disintegration time (DT) apparatus, Model No 121-P, Galvano Scientific (Pvt.) Ltd., Pakistan. For assessing the viscosity, the Lamy Rheology B-One Plus Viscometer, France was used. For developing the analytical method, a UV-spectrophotometer model NoUV-1800 by Shimadzu, Japan was used. The dissolution test was performed on Modified Dissolution Time Apparatus-USP Model No GDT-7L, Galvano Scientific (Pvt.) Ltd., Pakistan. Integrated Ultrasonic Homogenizer, SinoSonic, China was used for tissue homogenization.

### 2.3 Animals

Four-week-old female albino mice (BALB/c) bred at the Animal House of Shifa College of Pharmaceutical Sciences, Shifa Tameer-e-Millat University, Pakistan, were used for animal studies. The experimental group animals were divided and placed in separate cages under controlled temperature conditions, i.e., 25°C with a 12-h light-dark alternate cycle. *Ad libitum* diet was provided along with water. The *in vivo* experimental protocol was approved vide letter no. 1191-467-2018 Institutional Review Board and Ethics Committee, Shifa Tameer-e-Millat University, Islamabad, Pakistan.

### 2.4 Preparation of the formulation

Five different formulations referred herein onward as AC1, AC2, ACX1, ACX2, and ACX3, which were developed using only Carbopol 934 and HPMCK-15M as primary polymers and xanthan gum as a secondary polymer. AC1 and AC2 containing Carbopol-934 1.00 and 1.20 g, respectively, were soaked in 20 ml reverse osmosis (RO) water for 30 min at 70°C. Thereafter, 2.00 g of HPMC K-15 M and 0.200 g of methyl paraben sodium were added to 20 ml RO water at 70°C and mixed thoroughly with the primary polymer to achieve homogeneity. ADP, mixed with propylene glycol, was introduced into the system to prepare a homogenized mixture. RO water was added to make up the volume to 100 ml, and pH of the formulation was adjusted within the range of 4.0–4.5 by using NaOH/HCl.

The remaining three formulations (ACX1, ACX2, and ACX3) were prepared using three different combinations of Carbopol 934, HPMCK-15M, and xanthan gum. ACX1, ACX2, and ACX3 containing 1.00, 1.20, and 1.20 g Carbopol 934 and 0.50, 0.50, and 0.60 g xanthan gum, respectively, were formulated after keeping both the polymers soaked in 20 ml RO water at 70°C for 30 min. HPMC K-15 M 2.00 g and methyl paraben sodium 0.200 g suspended in RO at 70°C were added with the polymers. All the ingredients were homogenized. Lastly, ADP mixed with propylene glycol was added to the excipient mixture. The pH of the formulation was adjusted within the range of 4.0–4.5 by using NaOH/HCl. The composition of the prepared formulations has been summarized in Table 1.

**TABLE 1:** Percentage composition of tested formulations.

Ingredient	Formulation (%)				
	AC1	AC2	ACX1	ACX2	ACX3
Adapalene	1.00	1.00	1.00	1.00	1.00
Tetrahydrofuran	5.00	5.00	5.00	5.00	5.00
Carbapol 934	1.00	1.20	1.00	1.20	1.20
Xanthan gum	0.00	0.00	0.50	0.50	0.60
HPMC-K15M	2.00	2.00	2.00	2.00	2.00
Propylene glycol	10.0	10.0	10.0	10.0	10.0
Methyl paraben sodium	0.20	0.20	0.20	0.20	0.20
Maylake Blue # 1	0.002	0.002	0.002	0.002	0.002
HCl 10% solution	6.00	6.00	6.00	6.00	6.00
RO water	74.8	74.6	74.3	74.1	74.0

### 2.5 Formulation optimization

The experimental results were utilized to optimize the formulation by using the software Design Expert version 12 (Hooda et al., 2012; Solanki and Shah, 2016). Response surface methodology was applied with a 3-level quadratic Box–Behnken Design. The model was selected on recommendations of the software Wizard. The concentrations of Carbopol 934, HPMCK-15M, and xanthan gum were selected as variable factors having numeric values. The responses on which the effect of factors was studied were adhesiveness, spreadability, and viscosity. The experimental values were used to obtain an optimized prediction of the formulation.

### 2.6 Physicochemical analysis of formulation

In order to have a real-time comparison of the rheo-mechanical properties, a commercially available Metni-V intravaginal mucoadhesive gel, manufactured by M/s Shaigan Pharmaceuticals (Pvt) Ltd., Pakistan, was purchased from a local pharmacy.

#### 2.6.1 Adhesive nature

To assess the adhesive nature of formulations, solidified agar plates of 1.5% w/w at pH 4.5 adjusted using citric buffer to simulate vaginal conditions were used. Each sample (80 mg) was applied on an agar plate and 50 g weight was placed on top of it for 1 minute. Afterward, the agar plate was fixed in the disintegration time apparatus (DT) Model No 121-P, Galvano Scientific (Pvt.) Ltd., Pakistan, in citrate buffer at 37°C ± 1°C. The adherence time of each formulation was noted.

#### 2.6.2 Spreadability

To evaluate the spreadability of formulations, a test sample (1 g) was applied on a grid-printed glass plate and pressed with another glass plate and kept at 37°C. A weight of 100 g was placed on top of it for 1 min. The spreadability of test samples was measured in diameter (mm) on the printed-grid glass plate.

#### 2.6.3 Leakage

A leakage test was performed by diluting the sample (2 g formulation) in 0.75 ml citrate buffer considering that intravaginal fluid volume is approximately 0.6 ml, and on average, the intravaginal drug application is around 2–5 ml (Andrade et al., 2014). The diluted formulation was kept at 37°C for 24 h. Later, 80 mg of the diluted sample was transferred to a solidified 1.5% w/w agar glass slide. The glass slide was placed at 90° in a glass cylinder and placed in a water bath at 37°C for 2 h (Andrade et al., 2014). The distance covered by the diluted formulation was noted in millimeter.

#### 2.6.4 Viscosity and torque

Rheological studies, to estimate the viscosity of the formulations, were performed on a Lamy Rheology B-One Plus Viscometer, France, using L-4 and R-3 stainless steel spindle systems at 50 RPM for 30 s at 37°C. To establish a temperature equilibrium, the formulations were placed with the spindles for 5 min prior to the measurements.

### 2.7 Analytical method development

#### 2.7.1 Experimental requirements

A working standard of 100 mg ADP was accurately weighed and transferred to a 100-ml volumetric flask. It was later dissolved in tetrahydrofuran (THF), and the same was used to make up the volume to 100 ml. A 2-ml volume of the standard solution was transferred to a 100-ml volumetric flask. THF was used to make up the volume. Based on the results from the physical testing and the fact that it contained the excipients in the largest quantity, a suitable challenging candidate formulation ACX3 was used for developing and validating the assay method. A gel (2 g) equivalent to 20 mg of ADP was used for testing. The drug was transferred to a 100-ml volumetric flask, and THF was added to make up the volume to 100 ml. The final dilution was prepared by the abovementioned method at 0.02 mg/ml concentration. All the measurements for the purpose of the assay were taken at 321 nm.

#### 2.7.2 Validation of UV spectroscopy assay

In order to validate the analytical method for its performance, parameters including specificity, linearity and range, accuracy, precision, and ruggedness were assessed in accordance with the ICH guidelines for analytical method validation.

##### 2.7.2.1 Specificity

The specificity of the method was determined to establish that there was no interference from the excipients. The spectra of standard solutions, diluent (blank), placebo, and the sample were analyzed to evaluate the specificity of the method.

##### 2.7.2.2 Linearity and range

The linearity of an assay indicates that the signal (absorbance) is directly proportional to the concentration of the analyte in a tested sample. It was assessed by correlating the absorbance of triplicates of five ascending concentrations of ADP that were 10 , 15 , 20 , 25 , and 30 μg/ml. The corresponding absorbance values were plotted to calculate the regression. The acceptable value of correlation coefficient *r* ^2^ > 0.997 was considered.

##### 2.7.2.3 Accuracy

The accuracy signifies the degree of precision to which an analytical method correctly determines the amount of an analyte in a tested sample. It is determined by calculating the percentage recovery or %R. To ascertain the accuracy, the triplicates of four different concentrations i.e., 10 , 15 , 20, and 25 μg/ml were analyzed on a UV-spectrophotometer using the developed method. The percentage recovery was calculated using the following equation while setting the acceptable range for %R at 90–110%.

% Recovery=(Recovered conc. /Injected conc.) * 100

##### 2.7.2.4 Precision

Precision is the degree of similarity among individual tests performed using the developed method. The system precision was studied by running six replicates having concentrations of 20 μg/ml referred here on in as 100%. Moreover, the relative standard deviation (RSD) was calculated. The method precision to assess variation between days was performed by calculating the percentage assay for the standard solution prepared at a concentration 20 μg/ml and corresponding RSD values. The reference limit for acceptance was kept at < 2%.

##### 2.7.2.5 Ruggedness

Ruggedness of the method describes its capacity to remain unaffected by minor and deliberate variations. In the current study, three different analysts performed the experiment using three different wavelengths, with slight variations in the absorbance wavelength using triplicates prepared at 20 μg/ml on three different days, having slight difference in the experimental conditions with respect to temperature and relative humidity using the solutions prepared on different days.

### 2.8 *In vitro* dissolution studies

Dissolution studies were performed on modified dissolution time apparatus-USP Model No GDT-7L, Galvano Scientific (Pvt.) Ltd., Pakistan, to ascertain *in vitro* release of ADP from the developed formulation. Citrate buffer having 5% Tween 80 as the surface active agent was used as a medium to simulate the vaginal conditions. The modification in the apparatus was that it consisted of a 90-mm circular stand made up of 10 size stainless steel mesh fixed with 25-mm legs having immobilizers on the top to hold the glass slide for restraining the sample. The ADP gel (1 g) was placed on the glass slide with the temperature kept at 37 ± 0.5°C and a stirring speed of 50 RPM. The sample interval was kept at 5 min, and 5 ml of the sample was drawn six times with replenishment of fresh citrate buffer. The sample was filtered through a 0.45-micron membrane filter prior to the analysis on the UV-spectrophotometer. Dissolution studies were repeated thrice for calculating the mean drug release and reported as the percentage of drug.

### 2.9 *In vivo* absorption studies

To analyze the *in vivo* absorption of ADP, BALB/c mice were used. All animal testing was performed in accordance with the guidance laid in the Handbook of Good Laboratory Practice (GLP) quality practices for regulated non-clinical research and development by the World Health Organization (Research et al., 2010). Since the aim was to assess the tissue absorption of ADP from the developed formulation, the female BALB/c mice aged 28 days having an average weight of 15.5 g were used. The ADP mucoadhesive gel was administered following the application of local anesthetics, i.e., lignocaine. Approximately 200 mg of ADP 1% gel was administered into the vaginal cavity by pressing the mice downward from the tail base using two fingers. Three female mice were used for each of the total seven time points, starting at 15 min from administration of dose followed by 30, 45, 60, 90, 120, and 180 min. Upon reaching the defined time point, the mice were euthanized and dissected to isolate the cervix and vaginal tract. The tissue mass was transferred to a 5-ml solvent system and subjected to homogenization using a tissue homogenizer (integrated ultrasonic homogenizer, SinoSonic, China). The homogenized tissue mass was placed in a shaking bath for 12 h to make sure that all the drug in the tissue has been released. The mass was centrifuged at 8000 rpm to collect the supernatant, which was then passed through 0.45- and 0.22-micron filters in subsequent order.

## 3 Results

### 3.1 Adhesion, spreadability, and leakage

The experiments performed to assess the adhesion time revealed that formulations AC1 and AC2 containing one mucoadhesive agent, i.e., Carbopol-934 and HPMC K 15M, had the least adhesion time. The formulations having xanthan gum as an additional polymer performed better. The increase in the concentration of Carbolopol-934 enhanced the adhesion time in formulations ACX2 and ACX3 significantly. However, on increasing the concentration of xanthan gum, relatively, a small increase in the adhesion time was observed. The results of the adhesion test revealed that products ACX2 and ACX3 had an average adhesion time of 715 and 720 s, respectively, better than that of the commercially available products having an adhesion time of 672 s.

With regards to the spreadability of the formulations, nearly all the formulations had comparable spread diameter in comparison to that of the reference product (38 mm). AC1 and AC2 recorded a spread of 41 and 39 mm, respectively, while gels ACX1-3 had slight less average spreadability that was recorded at 39, 37, and 37 mm, respectively. This implied an inverse relation between the concentration of polymers and the diameter of the spread.

The tendency of the gel to leak upon dilution in the vaginal environment was assessed by the leakage test. Gels AC1 and AC2 travelled 47 and 43 mm, respectively, in comparison to the gels ACX1-3 that travelled 41, 39, and 35 mm, respectively. The reference formulation travelled 38 mm on the scale. The results from the rheo-mechanical experiments have been summarized in Table 2.

**TABLE 2:** Summary of rheo-mechanical properties of the formulations (mean ± SD; *n* = 3).

Formulation	Adhesion time (seconds)	Diameter (mm)	Distance travelled (mm)	Viscosity (cP)	Torque (m.N.m)
Ref. (Metni-V)	672 ± 2.45	38 ± 0.82	38 ± 0.82	98534.915 ± 0.41^a^	0.558 ± 0.02^a^
				1290.410 ± 0.54^b^	0.463 ± 0.01^b^
AC1	493 ± 3.74	41 ± 2.16	47 ± 0.82	97223.110 ± 1.28^a^	0.545 ± 0.03^a^
				1270.449 ± 0.76^b^	0.451 ± 0.01^b^
AC2	507 ± 3.56	39 ± 0.82	43 ± 0.82	97259.983 ± 0.29^a^	0.550 ± 0.01^a^
				1272.287 ± 0.73^b^	0.462 ± 0.02^b^
ACX1	615 ± 2.16	39 ± 0.82	41 ± 0.82	97394.843 ± 0.69^a^	0.561 ± 0.02
				1285.331 ± 0.31^b^	0.456 ± 0.02^b^
ACX2	715 ± 2.45	37 ± 1.63	39 ± 2.45	97754.243 ± 0.89^a^	0.564 ± 0.02^a^
				1289.901 ± 0.76^b^	0.4670 ± 0.02^b^
ACX3	720 ± 2.94	37 ± 1.63	35 ± 1.25	98912.443 ± 0.59^a^	0.566 ± 0.01^a^
				1296.220 ± 1.26^b^	0.471 ± 0.01^b^

a Represents the viscosity and torque measured with L4 spindle.

b Represents the viscosity and torque measured with R3 spindle.

### 3.2 Viscosity and torque

The viscosity of the formulations was studied using two different spindles. With Spindle L4, it was observed to be between 97,223.11 and 98,912.44 cP, while for the R3, the results ranged between 1,270.45 and 1,296.22 cP. The same system was used to study the torque values, which were noted between 0.54 and 0.56 m.N.m and 0.45–0.47 m.N.m for L4 and R3 spindle, respectively. Table 2 provides the values for the tested formulations. The reference formulation’s average viscosity using L4 and R3 spindle was noted at 98,534.91 and 1,290.41 cP, respectively, and the average torque was 0.56 and 0.46, respectively.

### 3.3 Optimization of the formulation

**FIGURE 1:**
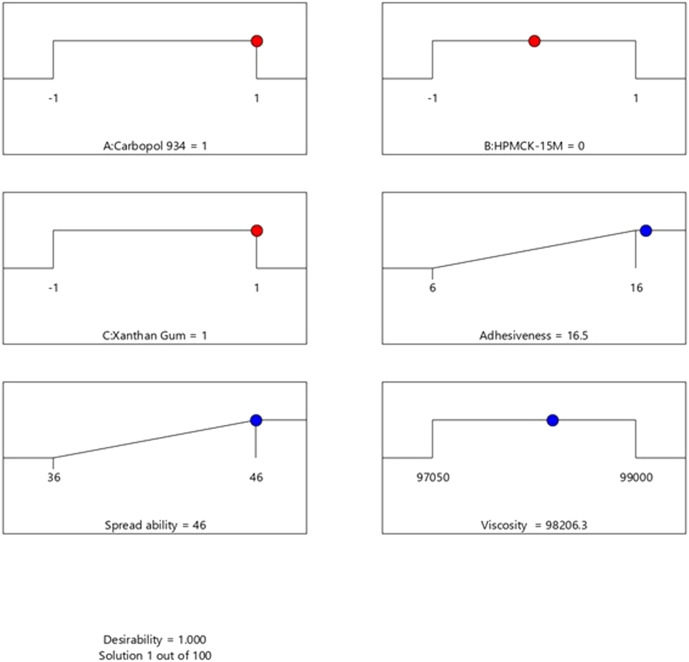
Optimization of the formulation by using Design Expert version 12. The analysis predicted 100 solutions for assessing and predicting the best formulation group. The solution with a desirability value of 1 indicated that an increase in the concentration of Carbopol 934 and xanthan gum would increase the spreadability and adhesiveness of the product along with the viscosity.

**FIGURE 2:**
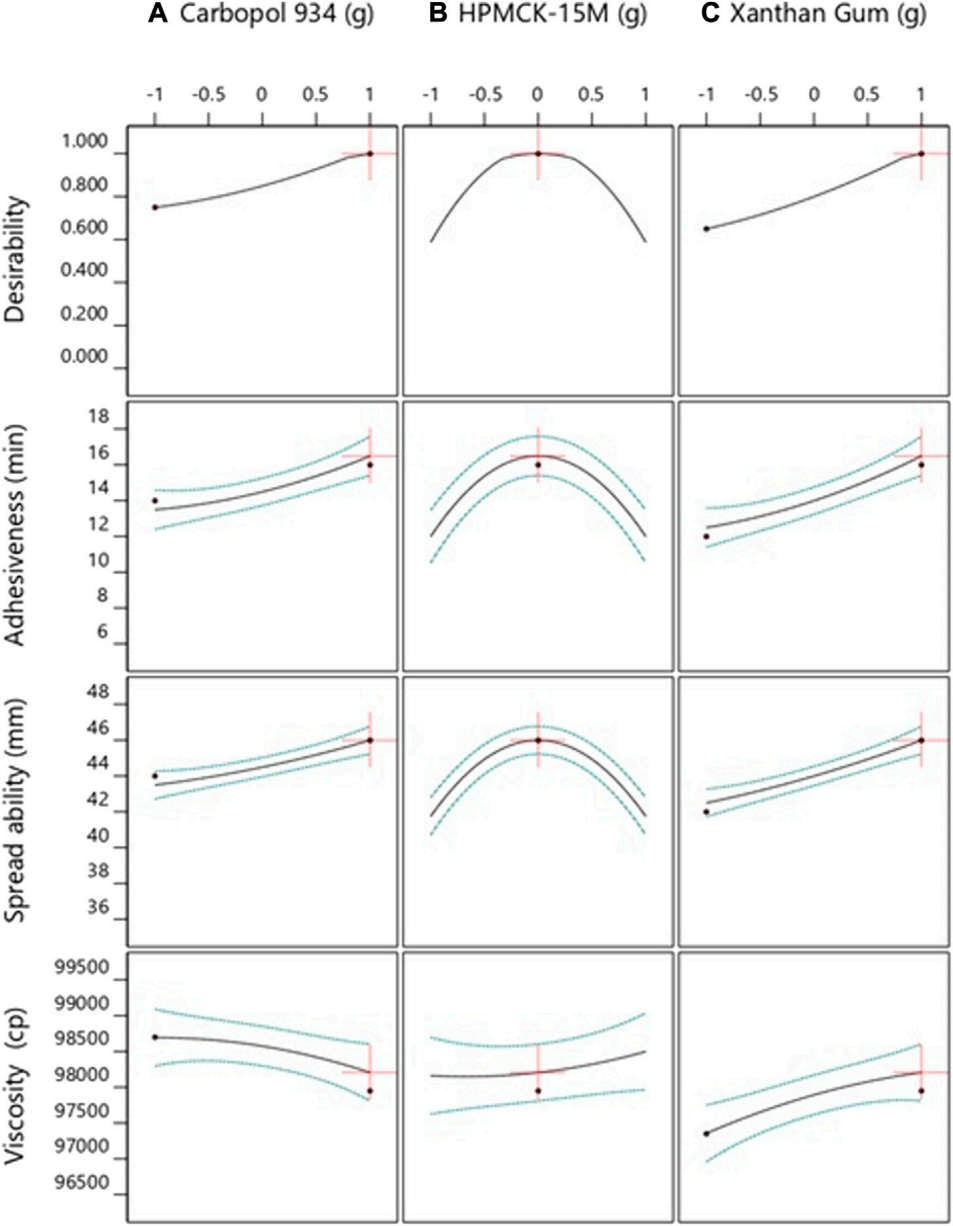
Representation of the relationship of various formulation factors and responses using Design Expert version 12. The factors included **(A)** Carbopol, **(B)** HPMCK-15M, and **(C)** xanthan gum, while the responses included adhesiveness, spreadability, and viscosity.

A total of 100 solutions were predicted using the computational benchmarking procedures for assessing if the developed formulations required further optimization or not. The solution with the highest desirability, i.e., 1, was indicative that increasing the concentration of Carbopol 934 and xanthan gum can increase the spreadability, adhesiveness, and viscosity of the product. The results showed that the formulations already developed are at their optimum level, and their further optimization was not practical. The optimization process and the results have been presented in Figure 1 and Figure 2 , respectively. The methodology adapted for the purpose has been summarized in Supplementary Tables S1–S3.

### 3.4 Validation of UV spectroscopy assay

The developed analytical method using UV-spectroscopy was subjected to various validation studies to ascertain the performance of the method for its specific, accurate, precise nature, linearity, range, recovery, and ruggedness for the formulation ACX3 as it had the maximum number and concentration of excipients and performed the best in the rheo-mechanical properties.

#### 3.4.1 Specificity

The acceptable criterion for the specificity of an experimental procedure is kept so that there is no interference with the solvent, blank, and placebo. From the placebo/diluent/blank spectra, it was concluded that no absorption was observed at the wavelength of 321 nm. Hence, for the tested samples, it can be concluded that there is no interference due to the placebo for the determination of ADP, thereby indicating that the method is selective and specific. The details have been provided in Supplementary Table S4.

#### 3.4.2 Linearity and range

The absorbance values of five concentrations of ADP were taken using the developed method. The calibration curve was plotted, and the coefficient of correlation (*r* ^2^) was found to be 0.9998, which lies within the acceptance criteria of not being less than 0.997. The calibration curve employed for the analysis of samples obtained from *in vitro* and *in vivo* analysis has been provided in Supplementary Figure S1. It can be concluded from the linearity and accuracy experiments that the analytical method for the assay of ADP Gel is linear and accurate across the range from 10 , 15 , 20, 25, and 30 μg/ml (50–150%) of the test concentration. Table 3 presents the results of the analysis.

**TABLE 3:** Linearity and range of the assay method.

Sample conc. (µg/ml)	10	15	20	25	30
Result of Rep 1 (%)	50.12	77.46	102.11	125.96	150.33
Result of Rep 2 (%)	50.64	75.92	100.35	126.24	151.09
Result of Rep 3 (%)	50.80	76.89	99.64	124.48	149.87
Mean (%)	50.52	76.75	100.70	125.56	150.43
Coefficient correlation (r)	0.99992				
Coefficient of determination (*r* ^2^)	0.9998				
Acceptance criteria	NLT:0.997				

#### 3.4.3 Accuracy and recovery

The accuracy and recovery of the assay method was determined using the equation for calculating the % R, which came out to be well within the acceptable limit of 90–110%. The assayed concentrations were 10, 15, 20, and 25 μg/ml for which the %R was found to be 101.77, 100.64, 100.34, and 100.42%, respectively. The results are summarized in Supplementary Table S5.

#### 3.4.4 Method and system precision

The system precision studies were performed by running six replicates of the standard solution having 100% assayed concentration. The RSD was calculated to be 0.495%. The method precision to measure the difference between days was performed, and the respective RSD values were calculated. These have been summarized in Supplementary Table S6. The method precision results were also within the prescribed limit at 1.12, 1.25, and 0.412% for three consecutive days.

#### 3.4.5 Ruggedness

The intended changes including the laboratory conditions for the experiment, personnel, and wavelength of UV-spectrophotometer were considered to perform the ruggedness studies of the developed method. The % R was calculated to be in a range of 97.92–99.58%. The details have been compiled in Supplementary Table S7.

### 3.5 *In vitro* dissolution Study

**FIGURE 3:**
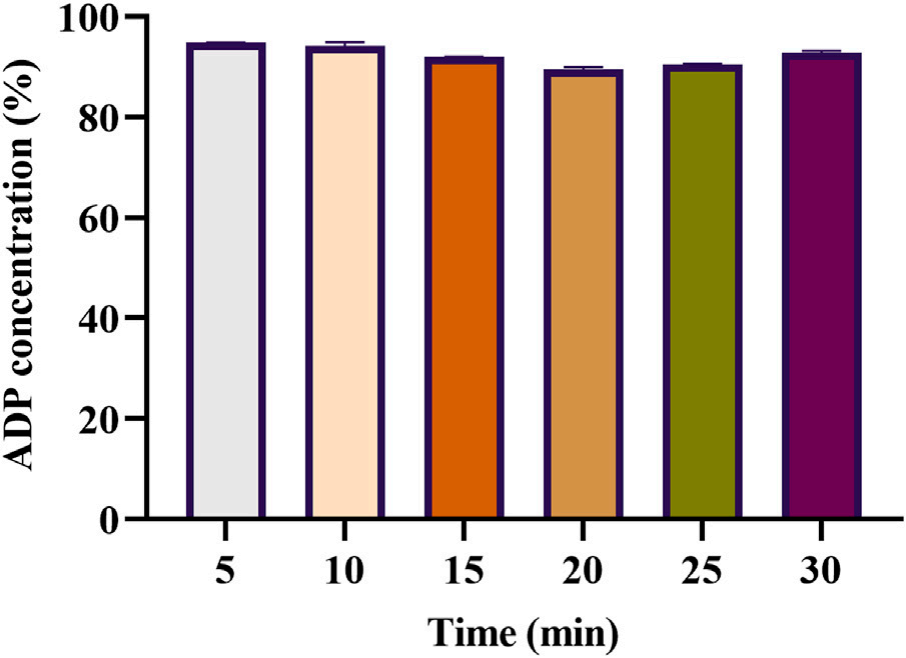
Graphical representation of the *in vitro* dissolution study for the assessment of the release of adapalene from the mucoadhesive vaginal gel (*n* = 3). The numeric values on the *y*-axis represent the percentage of dissolved adapalene (from the initial loaded dose 1 g of 1% adapalene muco-adhesive gel) and release pattern in the dissolution medium plotted against time.

For assessing *in vitro* drug release, 1 g ADP gel (ACX3) was placed on the glass slide of the modified USP-II dissolution apparatus (Weng and Parrott, 1983). Subsequent replenishment of buffer after each sample withdrawal was carried out. It was noted that 94.83% of the loaded ADP was detected in the first sample, followed by a gradual decrease in concentration up to 89.54% detected on the 4^th^ interval, and then a slight increase going up to 92.90% on completion of 30 min. The results have been summarized in Figure 3.

### 3.6 *In vivo* study

**FIGURE 4:**
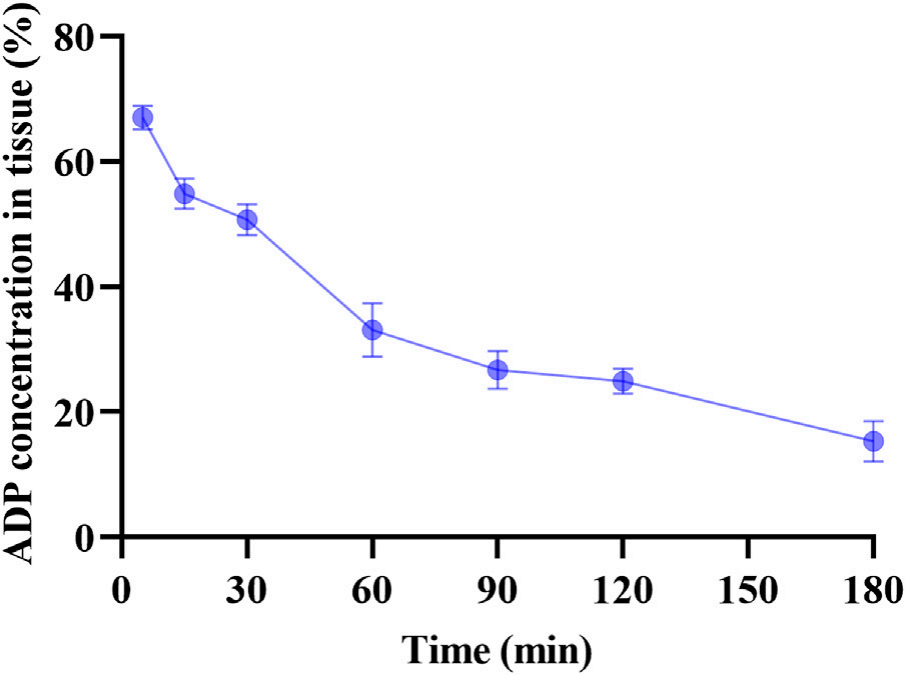
Assessment of *in vivo* absorption of adapalene from the developed mucoadhesive gel. The numeric values on the *y*-axis represent the average percentage of adapalene recovered from the cervical tissue of mice plotted against time (*n* = 3).

The cervical absorption of ADP from the mucoadhesive gel after *in vivo* experimentation on female mice was determined by analyzing the supernatant of the homogenized murine cervical tissue. The assay involved seven differently spaced time points after administering approximately 200 mg of ADP mucoadhesive gel (ACX3) through the mouse cervix. The absorbance (O.D.) values were translated into percentage absorption. The best tissue concentration having the mean percentage of 67% was detected at the first interval at 5 min from the first group of mice, followed by 54.8, 50.7, 33.1, 26.7, 24.9, and 15.3% at seven different time points, i.e., 15, 30, 60, 90, 120, and 180 min, respectively. The results have been summarized in Figure 4.

## 4 Discussion

Among the various diseases affecting the female reproductive tract, cervical cancer has the most severe clinical presentations as it is associated with both localized and systemic anatomical and physiological alterations. With more than half a million new cases and around 342,000 deaths annually, it is the fourth most common cause of cancer in women throughout the world (Sung et al., 2021). Moreover, 90% of these cases are reported in the LMIC, thereby indicating that alternate drug therapies need to be identified and developed against the disease. Drug repurposing can serve as a time-effective alternate to the laborious traditional drug discovery and development process. Drug molecules that are considered safe and effective in other related conditions can be repositioned for use in the targeted diseased condition. Adapalene (ADP) is one such molecule that has recently gained significant importance owing to its anti-inflammatory, anti-proliferative, anti-oxidant, and immunomodulatory effects, all of which are considered essential for an ideal antineoplastic agent (DiSilvestro et al., 2001; Rusu et al., 2020). The basic objective of the current study was to develop a mucoadhesive gel to ensure adequate absorption of ADP in the vaginal tissue, specifically the cervix.

The vaginal tissue provides a very challenging environment for formulation developers owing to variation in pH, temperature, age, sexual activities, and co-morbidities of the patients (Matteucci and Holmes, 2022). The mucus layer comprises enzymes, carbohydrates, lipids, nucleic acids, and salts that not only helps in providing defense against microbial infections but can also facilitate absorption of drug molecules in case they are delivered in adequate mucoadhesive formulations. Here, five formulations of ADP (AC1, AC2, ACX1, ACX2, and ACX3) were developed using different concentrations of the selected polymers, namely, Carbopol 934 and HPMC along with xanthan gum, which have already been reported to have mucoadhesive properties (Bilensoy et al., 2006; Andrade et al., 2014; Parvinroo et al., 2022). The polymers in the mucoadhesive formulations initially swell and physically connect to the mucin on the tissue followed by the strong chemical interaction of the polymer and the mucus membrane (Osmałek et al., 2021). It is after this point that the drug molecules, of sizes up to 300 kDa, easily penetrate into the cervical tissue. The formulations, therefore, are expected to possess efficient pharmaceutical attributes in order to achieve efficient pharmacotherapeutic targets. Considering the same, the prepared formulations were subjected to rheo-mechanical testing (Bachhav and Patravale, 2009). The results from the adhesion test suggested that using the three polymers together improve the adhesive nature of the product. A closer look points out that increasing the concentration of Carbopol 934 increases the adhesion time relatively more than that of xanthan gum or HPMC. The developed formulations exhibit better mucoadhesive potential than commercially available gels, suggesting that formulations having the three polymers together can adhere to vaginal mucosa for an appropriate time period above 10 min up to 12 min so that the drug can absorb efficiently. Another important characteristic for a mucoadhesive vaginal gel is its ability to spread upon application to ensure even coating of the vaginal mucosa. The results from the spreadability study suggest that the use of all the three polymers i.e., Carbopol-934, HMPC, and xanthan gum increase the intra-formulation interactions, resulting in decrease in the ability of the product to spread more upon application. However, the study results showed that the average spread diameter of all the formulations was comparable with the minimum reference product being 37 mm for ACX2 and ACX3 and maximum being 41 mm for AC1 compared to 38 mm for the reference formulation, therefore proposing the probable ease of application of the formulations. The leakage test was performed to ascertain the capacity of the formulation to withstand the dynamic vaginal environment. The results from the study show that all the formulations travelled comparable distance when studied along with the reference formulation. Furthermore, the formulations wherein three polymers were used performed better than the ones with two polymers, and the best performing formulation was ACX3 as it had covered the minimum distance during the rheo-mechanical studies. Hence, from the available data regarding the physical attributes of mucoadhesive gels in comparison to the reference formulations, the developed formulations performed well, signifying the use of co-polymers yielding better results, especially at higher concentrations (Andrade et al., 2014; Patil et al., 2019). The rheo-mechanical studies suggest that the formulations ACX2 and ACX3 are comparable to the commercially available mucoadhesive gel. Hence, the rationale for the potential of scaling up the formulation and its clinical use, further studies can be considered. Furthermore, the results from Design Expert prediction and optimization of the formulation with respect to the utilization of polymers suggest that formulations already developed are at their optimum level, and further tweaking may not be feasible. The polymers were selected to ensure that ideal mucoadhesion can be achieved. Carbopol-934 and HPMC, which are polyacrylate- and polycellulose-based agents, respectively, have been indicated in pharmaceutical use for development of oral, vaginal, and rectal dosage forms. HPMC has adequate solubility profile along with good swelling, hydration, thickening, and bioadhesive capabilities. Their inclusion in the formulation generally ensures a prolonged drug release as it controls the burst effect from the dosage form.

To ascertain that the formulation performs efficiently from the pharmaceutical stand point, an assay method was developed and validated for its analytical performance, followed by *in vitro* dissolution studies and *in vivo* absorption profiling of ADP in the cervical tissue (Pankti et al., 2013). The most challenging formulation scenario was considered with the rationale that the assay method developed for the formulation having the highest number and concentration of excipients would present the greatest challenge to the analytical method once developed. Therefore, product ACX3, which had performed well in the rheo-mechanical studies and had the highest number of excipients, was used for assay method validation. As any analytical method is required to be specific, the specificity was demonstrated by analyzing the spectra of the placebo formulation, diluent/blank at 321 nm, which showed no interference, indicating the high specificity of the method. The *r* ^2^ value was calculated at 0.9998, which is within the desired numeric value for the coefficient of correlation. Results on the analysis also suggested that the method operates within a useful range of 10–30 μg/ml. Hence, analyzing lower concentrations during the experimentation for dissolution and *in vivo* absorption will not pose to be an issue. The assay also exhibited accuracy within a range of 100 % ± 2%, across a range of concentrations, further advocating the suitability for its intended use. The system precision for the assay method was demonstrated as the RSD was calculated at 0.495%. The method precision studied over 3 consecutive days also demonstrated compliance to the set the limits. Finally, the assay was subjected to deliberate variation to see if the method can withstand the same. Since all these changes are expected to happen as the method will be used by different individuals on different equipment and under different laboratory conditions. The output after these deliberate changes were well within the limit. In summary, the results for assessing the validity of the assay method are developed from the aspects including accuracy, recovery, system and method precision, linearity, range, and ruggedness. The method is suitable for analyzing samples containing ADP since the method has been developed on the UV–Vis spectrometer, which is an acceptable technique for analytical method development and has additional benefits of being affordable, quick, and reliable. The inclusion of various validation parameters provides the confidence that the analytical method will have precise, accurate, and reproducible results for ADP mucoadhesive gel when analyzed using the method (Atole and Rajput, 2018).

In pursuit of predicting the pharmacokinetic properties of ADP delivered through the developed formulation, the release profile was determined in citrate buffer medium using a modified type-II USP dissolution apparatus (Weng and Parrott, 1983). Since, the intended route of application is intravaginal, citrate buffer was used which mimics the simulated vaginal fluid (Boyd and Hilas, 2014). Almost 95% of the loaded ADP from the mucoadhesive gel was dissolved and detected in the first sample taken after 5 min, followed by a slight decrease and then a gradual increase, and the results are suggestive of the fact that ADP release is quick and uniform. These pharmaceutical properties thereby strengthened the objective to develop an effective mucoadhesive formulation of ADP as it was capable of efficient drug delivery into the vaginal tract (Das Neves and Bahia, 2006; Liu et al., 2009). The final experiment in the current study was to evaluate the tissue absorption of ADP from the mucoadhesive gel in to the cervical tissue, which was performed in the murine model. Approximately, an average 67% of the drug was recovered from the murine cervical tissue after 5 minutes followed by a decrease up to 15.3% over a period of 180 min, endorsing the findings from earlier experiments, suggesting that the formulation is capable of delivering ADP efficiently into the cervix for localized effect. These properties correlated well with the pharmacological profiles reported for similar formulations earlier (Cevher et al., 2014; Aggarwal et al., 2017; Rençber et al., 2017).

## 5 Conclusion

The study has successfully demonstrated that ADP can be formulated as mucoadhesive vaginal gel having good tissue absorption using polymers such as Carbopol 934, HPMC-15M, and xanthan gum. The formulations having a combination of the three polymers performed similar or better than the commercially available vaginal gel when tested for their rheo-mechanical properties. The best performing formulation ACX3 presented an average adhesion time of 12 min, an average spread diameter of 37 mm, and an average distance travelled of 35 mm. The formulation in the dissolution studies showed dissolution up to 94.83% in the first 5 min and 67% recovery in the *in vivo* absorption studies, indicating toward potential of the adapalene mucoadhesive gel being scaled up for potential clinical use. Moreover, similar strategies can be adopted for development of mucoadhesive formulations of other pharmaceutical ingredients in order to ensure an efficient pharmacotherapeutic profile.

## Data Availability

The raw data supporting the conclusions of this article will be made available by the authors, without undue reservation.
