# Supplementary material for: Development of mucoadhesive adapalene gel for biotherapeutic delivery to vaginal tissue
Source: Front Pharmacol. 2022 Sep 29;13:1017549. doi: 10.3389/fphar.2022.1017549 (PMC9557122; doi:10.3389/fphar.2022.1017549)
Supplement: Supplementary file 1 [file Table1.DOCX]

Supplementary Table 1: Experimental design table using Design Expert v.12.0.

|  | Run | Factor 1 | Factor 2 | Factor 3 | Response 1 | Response 2 | Response 3 |
| --- | --- | --- | --- | --- | --- | --- | --- |
| Std |  | A:Carbopol 934 | B:HPMCK-15M | C:Xanthan Gum | Adhesiveness | Spread ability | Viscosity |
|  |  | (Grams) | (Grams) | (Grams) | (min) | (mm) | (cp) |
| 9 | 1 | 0 | -1 | -1 | 6 | 36 | 97050 |
| 13 | 2 | 0 | 0 | 0 | 12 | 42 | 98000 |
| 3 | 3 | -1 | 1 | 0 | 6 | 36 | 98000 |
| 1 | 4 | -1 | -1 | 0 | 6 | 36 | 97700 |
| 16 | 5 | 0 | 0 | 0 | 12 | 42 | 98000 |
| 2 | 6 | 1 | -1 | 0 | 10 | 40 | 98000 |
| 14 | 7 | 0 | 0 | 0 | 12 | 42 | 98000 |
| 10 | 8 | 0 | 1 | -1 | 6 | 36 | 97350 |
| 15 | 9 | 0 | 0 | 0 | 12 | 42 | 98000 |
| 6 | 10 | 1 | 0 | -1 | 12 | 42 | 97350 |
| 11 | 11 | 0 | -1 | 1 | 10 | 40 | 98650 |
| 4 | 12 | 1 | 1 | 0 | 10 | 40 | 98300 |
| 5 | 13 | -1 | 0 | -1 | 10 | 38 | 97050 |
| 12 | 14 | 0 | 1 | 1 | 10 | 40 | 99000 |
| 7 | 15 | -1 | 0 | 1 | 14 | 44 | 98700 |
| 17 | 16 | 0 | 0 | 0 | 12 | 42 | 98000 |
| 8 | 17 | 1 | 0 | 1 | 16 | 46 | 97950 |

| File Version | 12.0.3.0 |  |  |
| --- | --- | --- | --- |
| Study Type | Response Surface | Subtype | Randomized |
| Design Type | Box-Behnken | Runs | 17 |
| Design Model | Quadratic | Blocks | No Blocks |
| Build Time (ms) | 2.00 |  |  |

Supplementary Table 2: Factors included in Design Expert analysis

| **Factor** | **Name** | **Units** | **Type** | **Minimum** | **Maximum** | **Coded Low** | **Coded High** | **Mean** | **Std. Dev.** |
| --- | --- | --- | --- | --- | --- | --- | --- | --- | --- |
| A | Carbopol 934 | g | Numeric | -1.0000 | 1.0000 | -1 ↔ -1.00 | +1 ↔ 1.00 | 0.0000 | 0.7071 |
| B | HPMCK-15M | g | Numeric | -1.0000 | 1.0000 | -1 ↔ -1.00 | +1 ↔ 1.00 | 0.0000 | 0.7071 |
| C | Xanthan Gum | g | Numeric | -1.0000 | 1.0000 | -1 ↔ -1.00 | +1 ↔ 1.00 | 0.0000 | 0.7071 |

Supplementary Table 3: Responses included in Design Expert analysis

| **Response** | **Name** | **Units** | **Obs.** | **Analysis** | **Minimum** | **Maximum** | **Mean** | **Std. Dev.** | **Ratio** | **Transform** | **Model** |
| --- | --- | --- | --- | --- | --- | --- | --- | --- | --- | --- | --- |
| R1 | Adhesiveness | min | 17 | Polynomial | 6 | 16 | 10.35 | 2.94 | 2.67 | None | Quadratic |
| R2 | Spread ability | mm | 17 | Polynomial | 36 | 46 | 40.24 | 2.99 | 1.28 | None | Quadratic |
| R3 | Viscosity | cp | 17 | Polynomial | 97050 | 99000 | 97947.06 | 541.83 | 1.02 | None | Quadratic |

Supplementary Table 4: Determination of Specificity of the Assay

| **Adapalene Gel** | **Chromatograms** | **Comments** | **Absorbance** | |
| --- | --- | --- | --- | --- |
|  | **Diluent/Blank** | Blank shows no interference at absorbance point of the active ingredient. | Standard | Sample |
|  | **Standard** | Standard’s spectrum shows the 0.783 absorbance at 321nm. | 0.783 | 0.780 |
|  | **Placebo** | Placebo shows no interference near or at absorbance point of the active ingredient. |  |  |
|  | **Sample** | Sample’s spectrum shows 0.780 absorbance at 321nm and found similar to standard spectrum. |  |  |

Supplementary Table 5: Accuracy and recovery of the developed assay method

| Sample Conc. (%) | 10µg/ml | 15µg/ml | 20µg/ml | 25µg/ml |
| --- | --- | --- | --- | --- |
| Result of Rep. 1 | 50.34% | 75.44% | 100.70% | 126.11% |
| Result of Rep. 2 | 51.06% | 74.73% | 99.65% | 125.86% |
| Result of Rep. 3 | 49.40% | 76.27% | 100.69% | 124.64% |
| Mean | 50.26% | 75.48% | 100.34% | 125.53% |
| Std Dev | 0.8324 | 0.7707 | 0.6033 | 0.786 |
| Rel Std Dev (%) | 1.6559% | 1.021% | 0.6013% | 0.626% |
| %age Recovery | 101.77% | 100.64% | 100.34% | 100.42% |

Supplementary Table 6: Calculated precision of the developed assay method

| No. Of Replicates | | | System Precision | | | |
| --- | --- | --- | --- | --- | --- | --- |
| Replicate 1 | | | 101.73% | | | |
| Replicate 2 | | | 101.76% | | | |
| Replicate 3 | | | 102.48% | | | |
| Replicate 4 | | | 101.04% | | | |
| Replicate 5 | | | 101.40% | | | |
| Replicate 6 | | | 102.10% | | | |
| Mean | | | 101.75 | | | |
| Standard Dev | | | 0.506 | | | |
| Relative Standard Deviation | | | 0.495% | | | |
| Method Precision (within Days and between days variation) | | | | | | |
| Day | Sample | Assay | | Average Assay | STD | RSD |
| 1 | 1 | 101.41% | | 100.12% | 1.13 | 1.12% |
|  | 2 | 99.65% | |  |  |  |
|  | 3 | 99.30% | |  |  |  |
| 2 | 1 | 100.35% | | 100.00% | 1.25 | 1.25% |
|  | 2 | 101.05% | |  |  |  |
|  | 3 | 98.61% | |  |  |  |
| 3 | 1 | 98.94% | | 99.17% | 0.409 | 0.412% |
|  | 2 | 99.65% | |  |  |  |
|  | 3 | 98.94% | |  |  |  |

Supplementary Table 7: Ruggedness data for the developed assay

| Day | Wavelength | Sample | Assay Content  (Fresh. | Av. Assay Content  (Fresh) | Assay Content (Aged) | Av. Assay Content  (Aged) | Response (%) |
| --- | --- | --- | --- | --- | --- | --- | --- |
| 1 | 321.0nm | 1 | 99.30% | 100.70% | 98.96% | 98.61% | 97.92% |
|  |  | 2 | 102.12% |  | 97.91% |  |  |
|  |  | 3 | 100.70% |  | 98.96% |  |  |
| 2 | 322.0nm | 1 | 100.20% | 100.43% | 100.15% | 100.01% | 99.58% |
|  |  | 2 | 101.25% |  | 99.65% |  |  |
|  |  | 3 | 99.84% |  | 100.23% |  |  |
| 3 | 320.0nm | 1 | 100.26% | 101.16% | 99.80% | 99.87% | 98.72% |
|  |  | 2 | 101.68% |  | 99.34% |  |  |
|  |  | 3 | 101.56% |  | 100.47% |  |  |

**
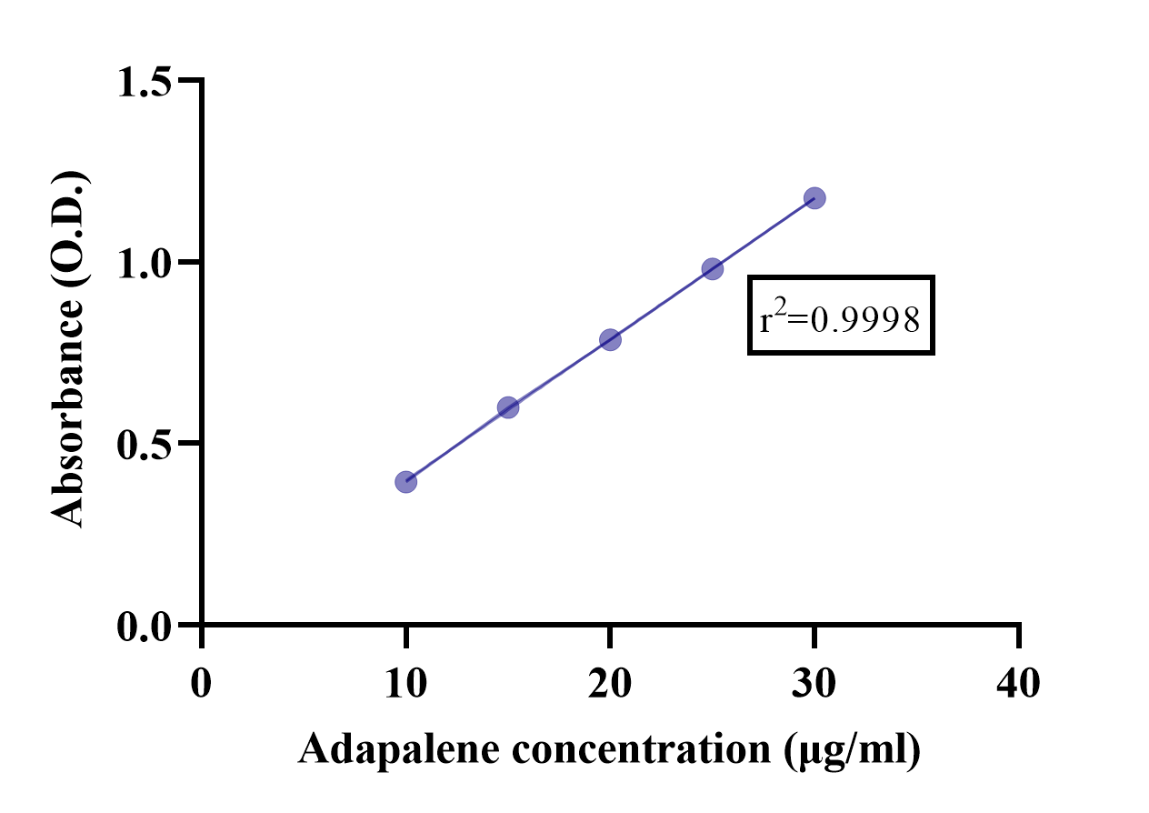
**

Supplementary Figure 1: Calibration curve of adapalene ranging from 10 to 30 µg/ml in mobile phase
