# Supplementary figures and images for: Development of mucoadhesive adapalene gel for biotherapeutic delivery to vaginal tissue
Source: Front Pharmacol. 2022 Sep 29;13:1017549. doi: 10.3389/fphar.2022.1017549 (PMC9557122; doi:10.3389/fphar.2022.1017549)

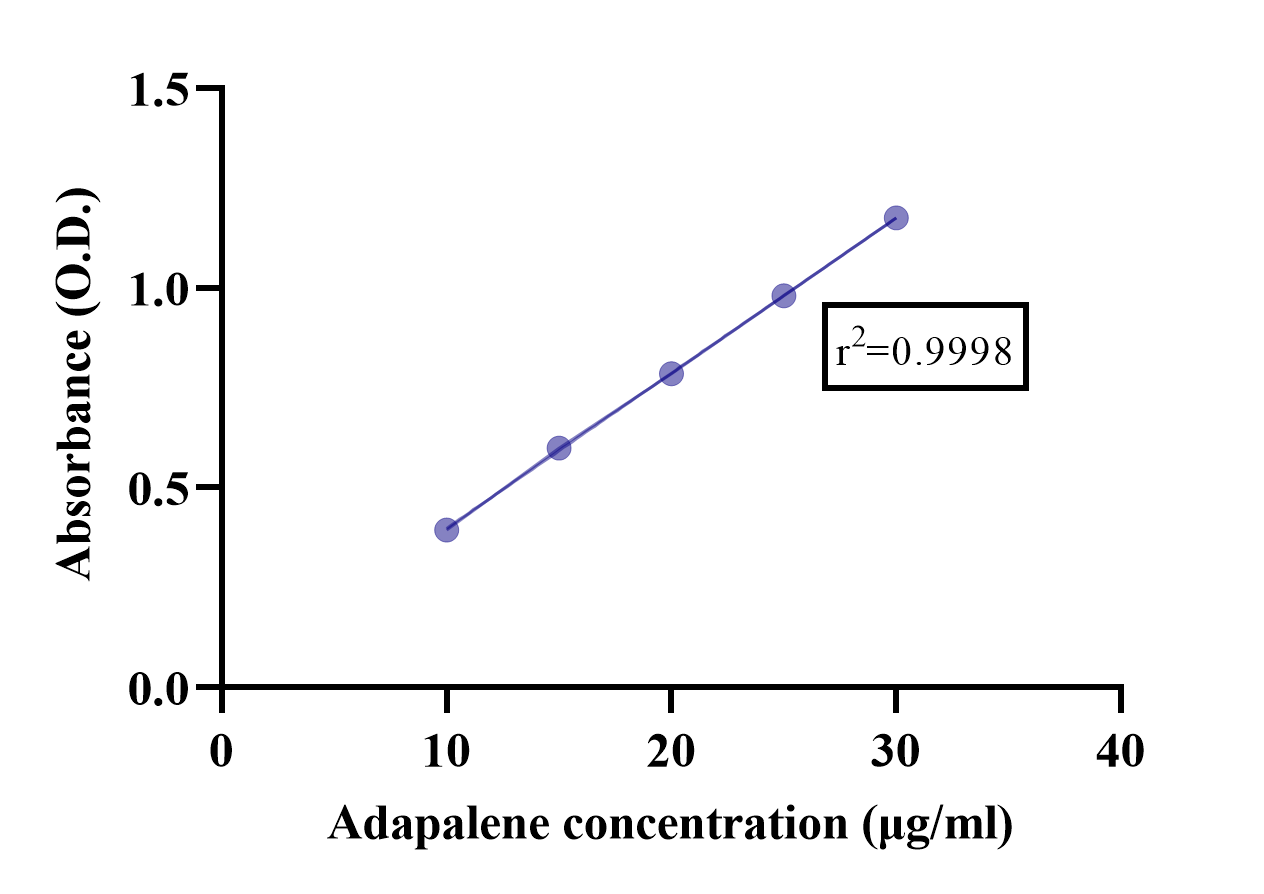

Supplement: Supplementary file 2 [file Image1.TIF]
